# A comparative study of efficacy and safety of transarterial chemoembolization with CalliSpheres and conventional transarterial chemoembolization in treating unresectable intrahepatic cholangiocarcinoma patients

**DOI:** 10.7150/jca.67523

**Published:** 2022-01-24

**Authors:** Tao Sun, Weihua Zhang, Lei Chen, Yanqiao Ren, Yiming Liu, Chuansheng Zheng

**Affiliations:** 1 Department of Radiology, Union Hospital, Tongji Medical College, Huazhong University of Science and Technology, Wuhan 430022, China.; 2 Hubei Province Key Laboratory of Molecular Imaging, Wuhan 430022, China.; 3 Department of Radiology, Union Hospital, Tongji Medical College and Wuhan National Laboratory for Optoelectronics, Huazhong University of Science and Technology.

**Keywords:** Intrahepatic cholangiocarcinoma, drug-eluting beads transarterial chemoembolization, overall survival, time to progression, treatment response

## Abstract

**Objective:** Previous studies reported that drug-eluting beads transarterial chemoembolization (DEB-TACE) with CalliSpheres is effective and safe to treat hepatocellular carcinoma patients and metastatic liver cancer patients, however few studies reported its clinical application in intrahepatic cholangiocarcinoma (ICC) patients. Therefore, this study aimed to compare the efficacy between DEB-TACE versus conventional transarterial chemoembolization (cTACE) in unresectable ICC patients.

**Methods:** Between January 2016 and June 2020, 89 patients with unresectable ICC were retrospectively analyzed, and enrolled into DEB-TACE group (N=40) and cTACE group (N=49) based on the transarterial treatment. Treatment response was assessed according to modified Response Evaluation Criteria in Solid Tumors (mRECIST). Time to progression (TTP) and overall survival (OS) were analyzed by using the Kaplan-Meier curve. Factors affecting OS and TTP were determined by Cox's proportional hazards regression model.

**Results:** DEB-TACE group showed higher DCR (87.5% vs. 65.3%, *P*=0.011), while similar ORR (67.5% vs. 57.1%, *P*=0.317) compared to cTACE group. Furthermore, DEB-TACE group had longer OS (median 10 months vs 6 months, *P*=0.006), while similar TTP compared to cTACE group (median 4 months vs 2 months, *P*=0.098). After adjustment by multivariant Cox's regression, DEB-TACE (versus cTACE) independently correlated with longer OS (*P*=0.031). Further subgroup analyses displayed that OS was prolonged in DEB-TACE group compared to cTACE group in patients with multiple tumors (*P*=0.032) and patients with no lymph node metastasis (*P*=0.023). Apart from abdominal pain, no difference of adverse events between the two groups was observed. There was no difference in liver function (Bilirubin, Albumin, Prothrombin time) before and after treatment (4 weeks) in both groups.

**Conclusion:** In patients with unresectable ICC, DEB-TACE significantly improved the OS when compared with cTACE and was well tolerated.

## Introduction

Intrahepatic cholangiocarcinoma (ICC) is a biliary epithelium malignancy with a rapid increase of global incidence rate and mortality rate over last several decades 1,16. However, Due to profound vascular invasiveness and distal metastasis, most ICC patients are in advanced stage when diagnosed and are not suitable for the curative options like surgical resection and transplant 1,3. Even after the curative options, early recurrence and metastasis were common. Therefore, it is a need to develop novel therapies to improve survival in unresectable ICC patients.

Conventional transarterial chemoembolization (cTACE) is the most common intra-arterial modality in treating unresectable ICC patients 5. However, minimal vascularity of the ICC and lower drug penetration due to loaded chemotherapeutic agent leakage remain huge concerns 6,7. To overcome these drawbacks, drug-eluting beads transarterial chemoembolization (DEB-TACE) is an alternative technique which uses a non-absorbable microsphere to embolize in the arteries of target tumor and to simultaneously release the loaded therapeutic agent in a controlled manner 6,7,8. In the past of 15 years, many drug-eluting beads (DEBs) have been produced, among which CalliSpheres microsphere is the first manufactured DEB in China with good loading and release profile 17. In terms of its clinical application, DEB-TACE with CalliSpheres microsphere has formerly been applied in treating hepatocellular carcinoma (HCC) patients and gained promising efficacy with less safety issues compared to cTACE 9,10,11. Furthermore, DEB-TACE with CalliSpheres microsphere also displays efficacy and safety in treating ICC patients 12,13. However, to our best knowledge, no comparison between DEB-TACE using CalliSpheres microsphere and cTACE has been conducted in patients with unresectable ICC yet. Therefore, we conducted this study and aimed to compare the efficacy and safety between DEB-TACE with CalliSpheres microsphere and cTACE in unresectable ICC patients.

## Methods

### Study design and population

This retrospective study was approved by Union Hospital, Tongji Medical College, Huazhong University of Science and Technology Review Board of our hospital. The informed consent from the patients were waive by the board because the study was a retrospective study. This study was conducted in accordance with the Declaration of Helsinki. A total of 89 patients with unresectable ICC treated by DEB-TACE or cTACE in our hospital between January 2016 and January 2019 were retrospectively analyzed. Clinical data of patients were reviewed, and the patients were considered eligible for analysis only if they met the following criteria: (i) diagnosed as unresectable ICC confirmed by liver biopsy (63 cases) or postoperative pathological examinations (26 cases); (ii) Child-Pugh stage A or B; (iii) Eastern Cooperative Oncology Group Performance Status (ECOG PS) score ≤1 point; (iv) treated by cTACE or DEB-TACE with CalliSpheres microsphere; (v) complete clinical data and survival data; (vi) without other malignancies. Among 89 ICC patients, 40 patients received DEB-TACE with CalliSpheres microsphere and were divided into DEB-TACE group; 49 patients underwent cTACE and were divided into cTACE group for the analysis. Written informed consents were collected from all the patients.

### Clinical data collection

For study analysis, the following preoperative clinical data were collected from the medical records systems: age, gender, hepatitis B virus (HBV) infection, ECOG PS score, Child-Pugh stage, tumor number, tumor size, lymph node metastasis, ascites, and biochemical indexes. In addition, TACE treatment information and previous treatments (such as surgery, and percutaneous transhepatic cholangial drainage/magnetic resonance cholangiopancreatography (PTCD/MRCP)) were also collected.

### TACE procedure

In brief, femoral artery puncture was performed by a micro-puncture system with a 5F vascular introducer (Cook, Bloomington, Indiana, USA). Celiac arteriography with or without superior mesenteric arteriography was implemented for evaluation of the feeding arteries of tumor, the arterial anatomy, as well as the patency status of the portal vein. Subsequently, superselective catheterization with a microcatheter was performed for the feeding arteries of tumor. The mixture of CalliSpheres microsphere (Jiangsu Hengrui Medicine Co. Ltd, Lianyungang, Jiangsu, China) and nonionic contrast medium (DEB-TACE group) or lipiodol (Lipiodol Ultrfluido, Guerbet, Paris, France) was injected into the tumor feeders through the microcatheter at a speed of 1 mL/min. In the DEB-TACE group, the CalliSpheres microsphere with diameter of 100-300 μm was used to load 80 mg epirubicin as chemoembolization agents. Drug-loading procedures of CalliSpheres microsphere were performed as the technological process described in previous study. In the cTACE group, 10-20 mL lipiodol (Lipiodol Ultrfluido, Guerbet, Paris, France) mixed with 20-40 mg epirubicin was used as chemoembolization agents. The injecting amount of mixture (CalliSpheres microsphere or lipiodol) was determined to the size of tumor. When the blood flow slowed or the small branch of portal vein appeared viewed by angiography, the TACE procedure stopped. The Polyvinyl Alcohol (PVA) could be added when it was necessary.

### Follow-up and assessments

The first follow-up was administered at fourth week following the first TACE procedure, during which, the abdominal contrast-enhanced computed tomography (CT), or magnetic resonance (MRI) was carried out for assessment of tumor response. Patients were subjected to another TACE if there were residual lesions or progression dieses (PD) revealed in the examination of CT or MRI. After the first follow-up, subsequent visit was conducted every 2 months, which was performed until January 2020. The documents of response evaluation and follow-up were collected, analyzed and compared. Evaluation of tumor response was in accordance with the modified RECIST criteria on the basis of the medical imaging of the abdominal enhanced CT or MRI. Objective response rates (ORR) and disease control rate (DCR) were calculated as reported in the previous study. Time to progression (TTP) was defined as the time interval from the first TACE treatment to the first disease progression. Overall survival (OS) was defined as the time interval from the first TACE treatment to patient's death regardless of the causes, or the last visit. In addition, adverse events were collected and reported according to the National Cancer Institute Common Terminology Criteria for Adverse Events (version 4.0).

### Statistical analysis

Data processing and analyzing were completed with the use of SPSS 24.0 (IBM, Armonk, New York, USA). For descriptive analysis, continuous variables and categorical variables were described as mean ± standard deviation (SD) and frequency (percentage), respectively. The Pearson χ^2^ test, correction χ^2^ test, Fisher's exact test, and independent-sample t test were applied to analyze the difference between two groups. Kaplan-Meier curve was plotted for displaying the profiles of accumulating TTP and OS. Estimation of difference in survival data between two groups was performed based on log-rank test. Variables with *P* value ≤0.2 in the univariate analysis were further included in the multivariate Cox's proportional hazards regression model analysis to assess the factors that affected TTP or OS. A *P* value <0.05 (two-tailed) was considered as there was statistical significance in corresponding analysis.

## Results

### Patients' clinical features

From January 2016 to June 2020, 89 unresectable ICC patients received DEB-TACE (40 patients) or cTACE (49 patients) were enrolled in this study. Baseline characteristics were well balanced between two groups and were listed in **Table 1**. The median follow-up period was 7 months (range, 2-19 months).

### Efficacy of DEB-TACE and cTACE for unresectable ICC

**Figure 2:**
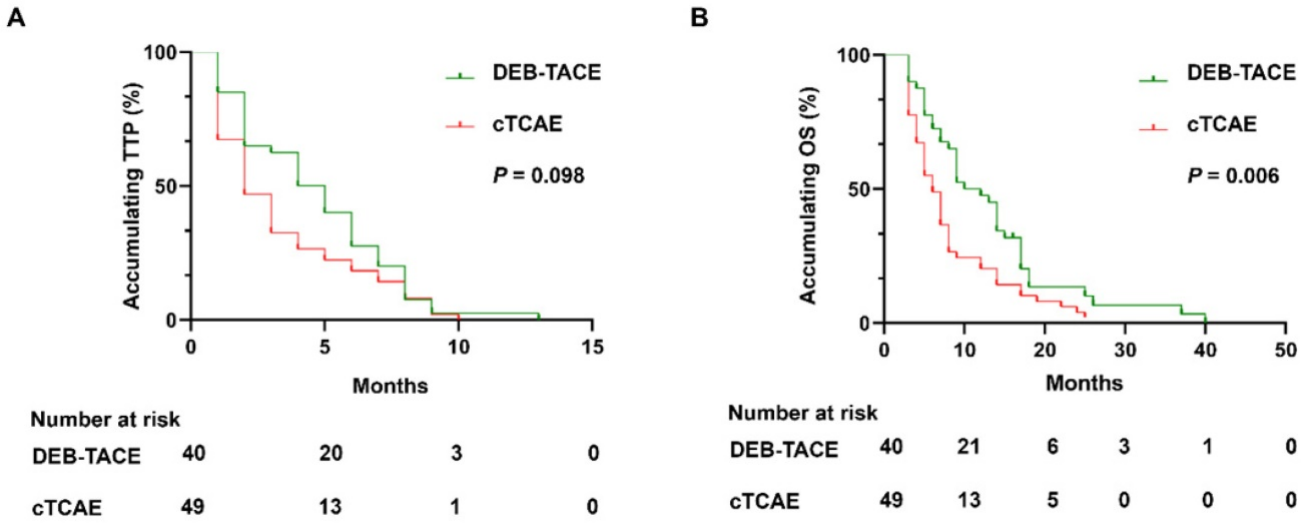
** DEB-TACE achieved longer OS but similar TTP compared to cTACE.** DEB-TACE achieved similar accumulating TTP (**A**), while prolonged OS (**B**) compared to cTACE. DEB-TACE: Drug-eluting beads transarterial chemoembolization, OS: overall survival, TTP: time to progression, cTACE: conventional transarterial chemoembolization, ICC: intrahepatic cholangiocarcinoma.

In DEB-TACE group, there were no case with CR, 27 (67.5%) cases with PR, 8 (20.0%) cases with SD, and 5 (12.5%) cases with PD (**Table 2**). In the cTACE group, there were no cases with CR, 28 (57.1%) cases with PR, 4 (8.2%) cases with SD, 18 (36.7%) cases with PD. The ORR of the DEB-TACE group was 67.5%, which was similar with the cTACE group (57.1%, *P*=0.317). However, the DCR of DEB-TACE was significantly higher than cTACE group (87.5% vs. 65.3%, *P*=0.011). During the follow-up, all patients died in the both groups. Although there was no difference of accumulating TTP between DEB-TACE group (median 4 months, 95% CI: (2.6, 5.3) months) and cTACE group (median 2 months, 95% CI: (1.2, 2.9) months) (*P*=0.098) (**Figure 2A**), DEB-TACE group (median 10 months, 95% CI: (6.1, 13.9) months) exhibited a longer accumulating OS compared to cTACE group (median 6 months, 95% CI: (4.5, 7.5) months) (*P*=0.006) (**Figure 2B**).

### Factors related to survival profile

Univariant Cox's regression model analysis displayed that treatment (DEB-TACE vs. cTACE) was not correlated with TTP in ICC patients (*P*=0.158) (**Table 3**). Furthermore, after adjustment by multivariant Cox's regression, treatment option (DEB-TACE vs. cTACE) failed to independently predict TTP in ICC patients (*P*=0.132). Univariant Cox's regression model analysis exhibited that treatment option (DEB-TACE vs. cTACE) correlated with longer OS in ICC patients (P=0.012) (**Table 4**). After adjustment by multivariant Cox's regression, treatment option (DEB-TACE vs. cTACE) could be an independent factor to predict longer OS in ICC patients (*P*=0.031).

Apart from that, univariate Cox's regression analysis revealed that multiple tumors, tumor size lymph node metastases, and previous treatments were related to TTP and OS. Multivariate Cox's regression analysis confirmed the results (**Table 3, Table 4**).

### Subgroup analysis

Tumor number (Single or Multiple) and lymph node metastasis (yes or no) were selected into subgroup analysis. The results showed that there was no difference of median TTP between DEB-TACE group and cTACE group in patients with single tumor (*P*=0.139), in patients with multiple tumors (*P*=0.370), in patients with lymph node metastasis (*P*=0.497) or in patients with no lymph node metastasis (*P*=0.051) (**Table 5**). In patients with multiple tumors or no lymph node metastasis, the median OS of DEB-TACE group was significantly higher than cTACE group (*P*=0.032,* P*=0.023, respectively). However, DEB-TACE group exhibited similar median OS to cTACE group in patients with single tumor (*P*=0.107) and in patients with lymph node metastasis (*P*=0.069).

### Comparison of adverse events

**Figure 3:**
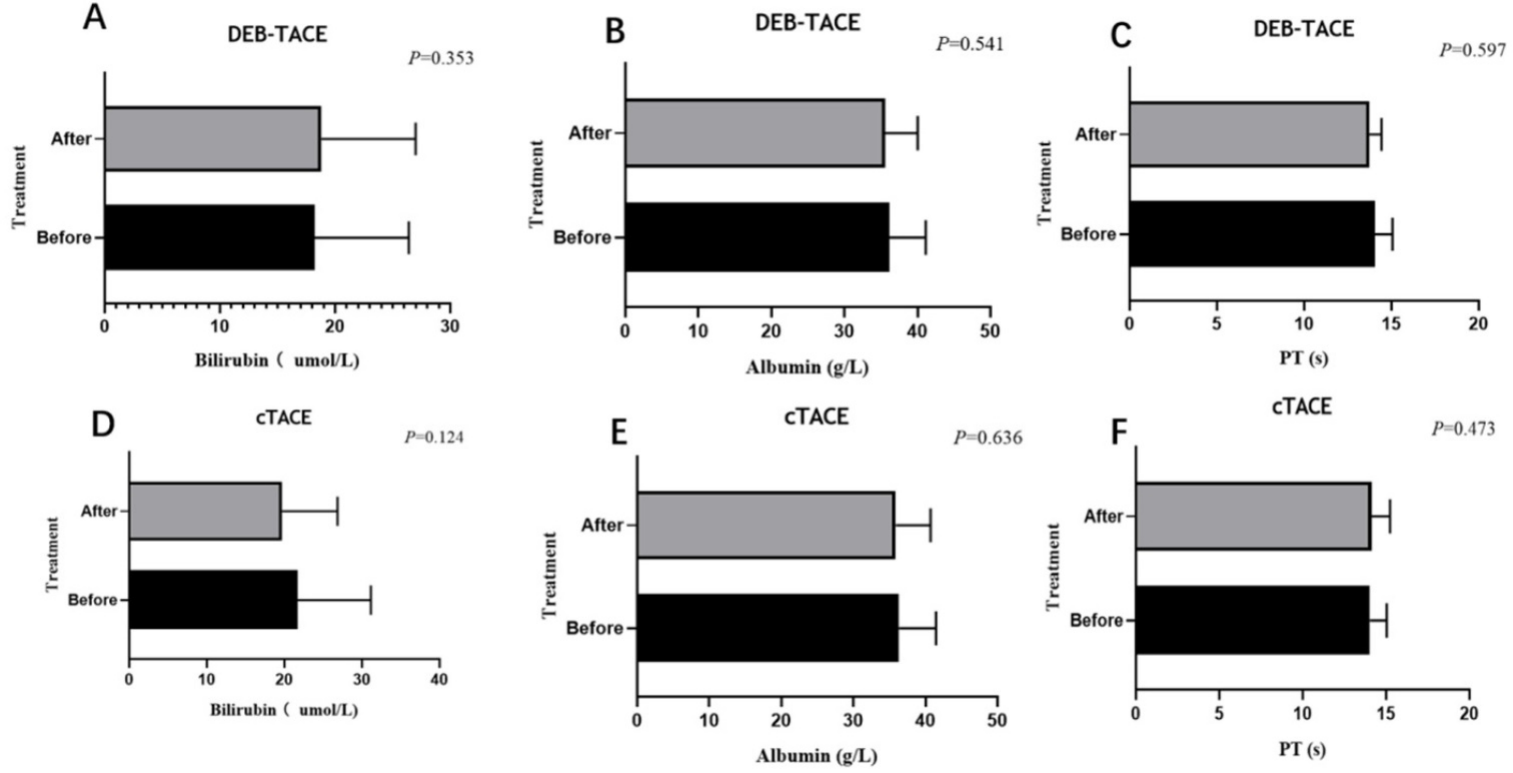
** Liver function before and after (4 weeks) the treatment.** Liver function including albumin (A, D), bilirubin (B, E), PT (C, F) of the DEB-TACE group or cTACE group before and after treatment at 4 weeks. Paired sample T-test showed no statistic difference (*P* > 0.05).

In DEB-TACE group, there were 2 (5.0%), 2 (5.0%) and 1 (2.5%) patient experienced Inguinal hematoma, hepatic arterial dissection and hepatorenal syndrome, respectively (**Table 6**). While in cTACE group, 2 (4.1%), 1 (2.0%) and 0 (0.0%) patients experienced Inguinal hematoma, hepatic arterial dissection and hepatorenal syndrome. There was no difference of adverse event rate between DEB-TACE group and cTACE group (all *P*>0.05). Liver function (bilirubin, albumin, and PT) were evaluated at 4 weeks after the first TACE (DEB-TACE, cTACE) treatment, and did not differ significantly from the baseline values at this time point (**Figure 3**).

## Discussion

DEB-TACE is widely used technique and gains huge attention in treating patients with liver malignancy 6. It is reported that DEB-TACE with Callispheres microsphere increases tumor response featured by higher ORR and CR rates compared to cTACE in HCC patients 9,10. Regarding its clinical application in ICC patients, two single-armed retrospective studies show that the ORR rates in DEB-TACE with Callispheres microsphere treated ICC patients are 65.9% and 67.6%, respectively 12,13. Since DEB-TACE with Callispheres microsphere displays improved efficacy and safe compared to cTACE in treating HCC patients, also DEB-TACE with Callispheres microsphere is safe and well-tolerant in ICC patients, therefore we hypothesized that DEB-TACE with Callispheres microsphere might achieve great efficacy compared to cTACE in ICC patients. However, no relevant study has been conducted. Thus, we performed this study and discovered that DEB-TACE with Callispheres microsphere therapy resulted in higher DCR compared to cTACE in treating ICC patients which could be explained as follows: DEB-TACE increased intratumoural concentration and released loaded chemotherapeutic agent in a controlled manner, therefore resulted in increased target tumor necrosis and further led to enhanced tumor response (featured by DCR in the present study) in ICC patients 6,7,8.

Apart from the comparison of DEB-TACE with Callispheres microsphere versus cTACE in term of their short-term efficacy, the comparison of these two TACE techniques regarding the long-term survival profile is also of great interest. However, no relevant study has been performed. In the present study, we discovered that DEB-TACE with Callispheres microsphere resulted in prolonged OS in ICC patients compared to cTACE, also it could independently predict longer OS. Furthermore, DEB-TACE with Callispheres microsphere achieved longer OS compared to cTACE in ICC patients with multiple tumors and patients with no lymph node metastasis. The possible reasons to explain these results were: (a) DEB-TACE increased tumor necrosis and enhanced tumor response as mentioned earlier, therefore led to reduced recurrence risk and favorable survival profile in ICC patients 6,7,8. (b) Regarding multiple tumors, it might indicate various cellular origins and molecular evolutions, which made them difficult to treat 15. While by using DEB-TACE, multiple tumors were easier to embolism compared with cTACE, which led to favorable prognosis in ICC patients 6. (c) Regarding lymph node metastasis, it suggested distal tumor formation and growth through the lymphatic vessels, therefore led to more advanced stage of ICC and increased mortality risk, which eventually resulted in unfavorable survival profile in ICC patients 14. Taken together, ICC patients underwent DEB-TACE with Callispheres microsphere achieved favorable survival profile than cTACE.

In terms of the safety profile of DEB-TACE with Callispheres microsphere in ICC patients, one study discovers that the major adverse events of DEB-TACE with Callispheres microsphere in ICC patients are abdominal pain, fever, vomiting and nausea, which are all mild and manageable 12. Among these, patients received DEB-TACE significantly less likely to experience abdominal pain compared to patients received cTACE. Another single-armed retrospective study shows that DEB-TACE with Callispheres microsphere induces similar adverse events (including pain, fever, vomiting and nausea) in ICC patients, suggesting that DEB-TACE with Callispheres microsphere is relative safe in treating ICC patients 13. In this double-armed comparative study, we discovered that the main adverse events in ICC patients related to DEB-TACE with Callispheres microsphere were inguinal hematoma, hepatic arterial dissection and hepatorenal syndrome, suggesting the relative safe profile of DEB-TACE with Callispheres microsphere. Although the load of chemotherapy drugs in DEB-TACE is higher, there was no difference of adverse event rates between DEB-TACE with Callispheres microsphere and cTACE in ICC patients, which could be explained that DEB-TACE reduced systematic toxicity by preventing loaded antitumor agent leakage compared to cTACE 6,7,8.

Several limitations existed in the current study. Firstly, the sample size in our study was relatively small, therefore a further study with larger sample size to validate the safety and efficacy of DEB-TACE with Callispheres microsphere in unresectable ICC patients was necessary. Secondly, as a real-world study, some compounding factors might occur, therefore further randomized controlled trial (RCT) validation was needed. Finally, this was a single-center study, which might cause selection bias of patients, and further study with multiple centers was necessary.

## Conclusions

In conclusion, DEB-TACE with Callispheres microsphere is superior to cTACE in treating unresectable ICC patients regarding its better efficacy and equal safety.

## Figures and Tables

**Figure 1:**
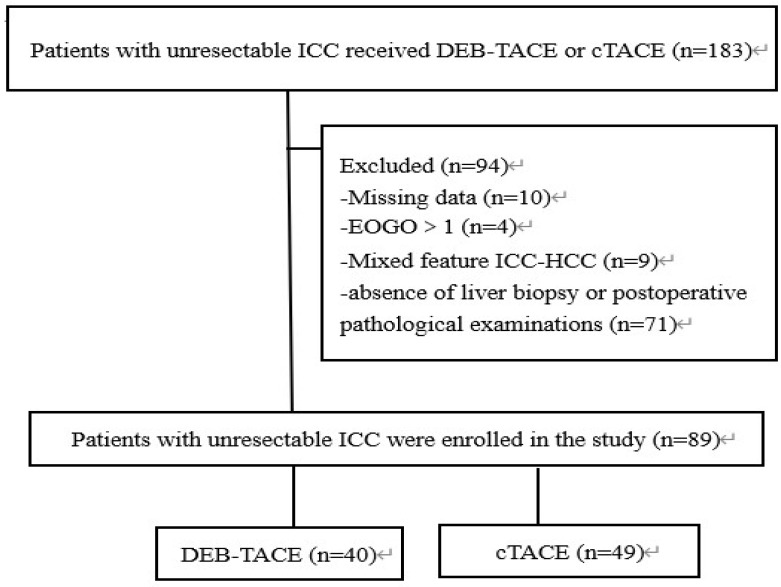
** Flow chart.** DEB-TACE: Drug-eluting beads transarterial chemoembolization, cTACE: conventional transarterial chemoembolization, ICC: intrahepatic cholangiocarcinoma.

**Table 1:** Characteristics of patients

Characteristics	DEB-TACE (N = 40)	cTACE (N = 49)	*P* value
Age (years), mean±SD	61.8±10.7	57.4±10.4	0.053
**Gender, No. (%)**			0.608
Male	25 (62.5)	28 (57.1)	
Female	15 (37.5)	21 (42.9)	
HBV, No. (%)	3 (7.5)	5 (10.2)	0.943
**ECOG PS score, No. (%)**			0.722
0	17 (42.5)	19 (38.8)	
1	23 (57.5)	30 (61.2)	
**Child-Pugh stage, No. (%)**			0.322
A	35 (87.5)	39 (79.6)	
B	5 (12.5)	10 (20.4)	
**Tumor number, No. (%)**			0.512
Single	16 (40.0)	23 (46.9)	
Multiple	24 (60.0)	26 (53.1)	
Tumor size (cm), mean±SD	7.9±6.6	7.0±4.1	0.410
Lymph node metastasis, No. (%)	31 (77.5)	29 (59.2)	0.744
Ascites, No. (%)	3 (7.5)	4 (8.2)	0.999
**Biochemical indexes**			
ALT (U/L), mean±SD	41.8±53.2	49.8±45.7	0.454
Albumin (g/L), mean±SD	35.5±5.1	35.8±10.5	0.375
Bilirubin (umol/L), mean±SD	19.8±6.3	19.3±4.7	0.452
PT (s) , mean±SD	13.9±2.1	13.7±1.9	0.534
Neutrophile (x10^9^/L), mean±SD	5.0±2.1	4.3±2.1	0.143
Lymphocyte (x10^9^/L), mean±SD	1.1±0.5	1.5±0.6	0.002
PLT (x10^9^/L), mean±SD	188.8±86.0	190.4±84.7	0.931
CA125 (U/mL), mean±SD	131.5±204.8	121.8±273.3	0.853
CA199 (U/mL), mean±SD	518.4±525.7	528.5±1722.0	0.972
**TACE session, No. (%)**			0.741
1	19 (47.5)	25 (51.0)	
≥2	21 (52.5)	24 (49.0)	
**Previous treatments**			
Surgery, No. (%)	8 (20.0)	18 (36.7)	0.084
Radiofrequency ablation, No. (%)	0(0)	0(0)	-
Systematic chemotherapy, No. (%)	4 (10.0)	6 (12.2)	0.739
Targetded therapy, No. (%)	0 (0)	0 (0)	-
PTCD/MRCP, No. (%)	9 (22.5)	15 (30.6)	0.391

DEB-TACE, drug-eluting beads transarterial chemoembolization; cTACE, conventional transarterial chemoembolization; SD, standard deviation; HBV, hepatitis B virus; ECOG PS, Eastern Cooperative Oncology Group Performance Status; ALT, alanine aminotransferase; PT, Prothrombin time; PLT, blood platelet; CA125, carbohydrate antigen 125; CA199, carbohydrate antigen 199; PTCD, percutaneous transhepatic cholangial drainage; MRCP, magnetic resonance cholangiopancreatography.

**Table 2:** Treatment response

Response	Patients, No. (%)		*P* value
	DEB-TACE (N = 40)	cTACE (N = 49)	
CR	0 (0.0)	0 (0.0)	
PR	27 (67.5)	28 (57.1)	
SD	8 (20.0)	4 (8.2)	
PD	5 (12.5)	18 (36.7)	
ORR	27 (67.5)	28 (57.1)	0.317
DCR	35 (87.5)	32 (65.3)	0.011

DEB-TACE, drug-eluting beads transarterial chemoembolization; cTACE, conventional transarterial chemoembolization; CR, complete response; PR, partial response; SD, stable disease; PD, progression disease; ORR, objective response rate; DCR, disease control rate.

**Table 3:** Univariate and multivariate Cox's regression analysis for TTP

Parameters	Univariate HR (95%CI)	*P* value	Multivariate HR^#^ (95%CI)	*P* value
Treatment (cTACE vs. DEB-TAEC)	1.356 (0.888, 2.072)	0.158	1.386 (0.907, 2.118)	0.132
Age	0.987 (0.967, 1.008)	0.225	-	-
Gender (Female vs. male)	1.011 (0.659, 1.550)	0.960	-	-
HBV (no vs. yes)	1.445 (0.658, 3.171)	0.359	-	-
ECOG PS score (1 vs. 0)	1.080 (0.701, 1.663)	0.728	-	-
Child-Pugh stage (B vs. A)	0.819 (0.461, 1.455)	0.496	-	-
Tumor number (multiple vs. single)	1.823 (0.733, 2.921)	0.034	1.819(0.742, 2.919)	0.039
Tumor size	1.706 (0.967, 1.048)	0.041	-	-
Lymph node metastases (yes vs. no)	1.732 (0.455, 3.179)	0.020	1.715 (0.445, 3.150)	0.047
Ascites (no vs. yes)	0.829 (0.382, 1.800)	0.635	-	-
ALT	0.999 (0.995, 1.004)	0.741	-	-
Neutrophile	0.992 (0.888, 1.109)	0.888	-	-
Lymphocyte	1.228 (0.830, 1.816)	0.303	-	-
PLT	1.001 (0.998, 1.003)	0.530	-	-
CA125	1.000 (0.999, 1.001)	0.571	-	-
CA199	1.000 (1.000, 1.000)	0.943	-	-
TACE session (≥2 vs. 1)	1.155 (0.758, 1.761)	0.501	-	-
Previous treatments (no vs. yes)	1.782 (0.680, 2.724)	0.039	1.453 (0.569, 2.349)	0.028
PTCD/MRCP (no vs. yes)	1.028 (0.652, 1.621)	0.906	-	-

#variables with *P* value ≤0.2 in the univariate analysis were further included in the multivariate Cox proportional hazards regression model analysis. TTP, time to progression; HR, hazard ratio; CI, confidence interval; cTACE, conventional transarterial chemoembolization; DEB-TACE, drug-eluting beads transarterial chemoembolization; HBV, hepatitis B virus; ECOG PS, Eastern Cooperative Oncology Group Performance Status; ALT, alanine aminotransferase; PLT, blood platelet; CA125, carbohydrate antigen 125; CA199, carbohydrate antigen 199; PTCD, percutaneous transhepatic cholangial drainage; MRCP, magnetic resonance cholangiopancreatography.

**Table 4:** Univariate and multivariate Cox's regression analysis for OS

Parameters	Univariate HR (95%CI)	*P* value	Multivariate HR^#^ (95%CI)	*P* value
Treatment (cTACE vs. DEB-TAEC)	1.763 (1.131, 2.749)	0.012	1.654 (1.048, 2.608)	0.031
Age	0.986 (0.966, 1.007)	0.203	-	-
Gender (Female vs. male)	1.031 (0.665, 1.599)	0.891	-	-
HBV (no vs. yes)	1.185 (0.540, 2.604)	0.672	-	-
ECOG PS score (1 vs. 0)	1.343 (0.859, 2.099)	0.196	1.326 (0.846, 2.078)	0.219
Child-Pugh stage (B vs. A)	0.929 (0.521, 1.658)	0.804	-	-
Tumor number (multiple vs. single)	1.997 (0.711, 2.993)	0.025	1.852 (0.690, 2.895)	0.037
Tumor size	1.820 (0.979, 2.563)	0.042	1.734(0.832, 2.498)	0.045
Lymph node metastases (no vs. yes)	0.829 (0.524, 1.312)	0.424	-	-
Ascites (no vs. yes)	0.704 (0.322, 1.539)	0.379	-	-
ALT	1.000 (0.996, 1.005)	0.825	-	-
Neutrophile	1.015 (0.907, 1.137)	0.792	-	-
Lymphocyte	1.311 (0.887, 1.937)	0.175	1.213 (0.808, 1.820)	0.352
PLT	1.001 (0.998, 1.003)	0.697	-	-
CA125	1.000 (0.999, 1.001)	0.645	-	-
CA199	1.000 (1.000, 1.000)	0.991	-	-
TACE session (≥2 vs. 1)	1.150 (0.748, 1.766)	0.524	-	-
Previous treatments (no vs. yes)	1.809 (0.751, 2.946)	0.034	1.794 (0.742, 2.894)	0.042
PTCD/MRCP (no vs. yes)	1.218 (0.756, 1.961)	0.417	-	-

#variables with *P* value ≤0.2 in the univariate analysis were further included in the multivariate Cox proportional hazards regression model analysis. OS, overall survival; HR, hazard ratio; CI, confidence interval; cTACE, conventional transarterial chemoembolization; DEB-TACE, drug-eluting beads transarterial chemoembolization; HBV, hepatitis B virus; ECOG PS, Eastern Cooperative Oncology Group Performance Status; ALT, alanine aminotransferase; PLT, blood platelet; CA125, carbohydrate antigen 125; CA199, carbohydrate antigen 199; PTCD, percutaneous transhepatic cholangial drainage; MRCP, magnetic resonance cholangiopancreatography.

**Table 5:** Subgroup analysis of TTP and OS based on tumor features

Items	DEB-TACE	cTACE	*P* value
**Single tumor**	n = 16	n = 23	
Median TTP (95%CI), months	6 (4.7, 7.3)	2 (0.7, 3.3)	0.139
Median OS (95%CI), months	14 (12.7, 15.3)	6 (4.0, 8.0)	0.107
**Multiple tumors**	n = 24	n = 26	
Median TTP (95%CI), months	4 (3.1, 4.9)	2 (1.0, 3.0)	0.370
Median OS (95%CI), months	9 (7.1, 10.9)	6 (4.3, 7.7)	0.032
Lymph node metastasis	n = 31	n = 29	
Median TTP (95%CI), months	4 (2.0, 6.0)	3 (2.1, 3.9)	0.497
Median OS (95%CI), months	9 (4.6, 13.4)	6 (4.2, 7.8)	0.069
No lymph node metastasis	n = 9	n = 20	
Median TTP (95%CI), months	6 (2.1, 8.9)	2 (0.6, 3.4)	0.051
Median OS (95%CI), months	17 (12.8, 21.2)	6 (3.8, 8.2)	0.023

TTP, time to progression; OS, overall survival; DEB-TACE, drug-eluting beads transarterial chemoembolization; cTACE, conventional transarterial chemoembolization.

**Table 6:** Adverse events after TACE treatment

Adverse events	DEB-TACE (N = 40)	c-TACE (N = 49)	*P* value
Vomiting/Nause	28 (70.0)	35 (71.4)	0.883
Abdominal pain	25 (62.5)	42 (85.7)	0.012
fever	16 (40.0)	26 (53.1)	0.220
Inguinal hematoma, No. (%)	2 (5.0)	2 (4.1)	0.836
Hepatic arterial dissection, No. (%)	2 (5.0)	1 (2.0)	0.441
Hepatorenal syndrome, No. (%)	1 (2.5)	0 (0.0)	0.204
Pulmonary oil embolization, No. (%)	0 (0.0)	0 (0.0)	-

TACE,transarterial chemoembolization; DEB-TACE, drug-eluting beads transarterial chemoembolization; cTACE, conventional transarterial chemoembolization.

## Abbreviations

ICC: intrahepatic Cholangiocarcinoma
DEB-TACE: drug-eluting beads transarterial chemoembolization
c-TACE: conventional TACE
TTP: the median time to progression
OS: overall survival
DCR: disease control rate
mRECIST: the modified Response Evaluation Criteria in Solid Tumors
HCC: hepatocellular carcinoma
ECOG: Eastern Cooperative Group Performance Status
CT: computed tomography
MRI: magnetic resonance image
CR: complete response
PR: partial response
SD: stable disease
PD: progressive disease
